# *Blastocystis* first detected in *Sciurus vulgaris* and *Sciurus vulgaris exalbidus* in Chengdu, China

**DOI:** 10.17221/71/2024-VETMED

**Published:** 2025-09-29

**Authors:** Xiaobo Li, Haocheng Huang, Yifan Liu, Wanyu Meng, Zhijun Zhong, Ziyao Zhou, Guangneng Peng, Jianbao Han, Haifeng Liu

**Affiliations:** ^1^Biosafety Laboratory of West China Hospital of Sichuan University, Chengdu, Sichuan, P.R. China; ^2^Department of Veterinary Surgery, College of Veterinary Medicine, Sichuan Agricultural University, Chengdu, Sichuan, P.R. China

**Keywords:** *Blastocystis* species, epidemiology, subtype 4, zoonotic transmission

## Abstract

The anaerobic unicellular protist *Blastocystis* is widely recognised for its presence in the gastrointestinal systems of humans and various animals globally. However, there is a paucity of reports on the prevalence and subtype (ST) distribution of *Blastocystis* in the squirrel population. This study was conducted to determine the prevalence and genetic diversity of *Blastocystis*, as well as its zoonotic potential, among *Sciurus vulgaris* and *Sciurus vulgaris exalbidus* in Chengdu, China. A total of 41 faecal samples (31 from *Sciurus vulgaris*, 10 from *Sciurus vulgaris exalbidus*) were analysed for the presence of *Blastocystis* sp. using the polymerase chain reaction (PCR) amplification of the small subunit ribosomal RNA (*SSU rRNA*) gene. Our findings revealed a positive rate of 4.88% (2/41 samples) for *Blastocystis* sp., with both identified as ST4 through nucleotide sequence homology and phylogenetic analysis. Given the zoonotic nature of this subtype, farmed squirrels may serve as potential reservoirs for *Blastocystis* transmission to humans and domestic animals. These findings are essential for developing effective control strategies against *Blastocystis* in the study region and enhancing our comprehension of the genetic spectrum of *Blastocystis* within *Sciurus vulgaris* and *Sciurus vulgaris exalbidus*.

*Blastocystis*, a prevalent intestinal eukaryote, has been detected in humans and many animal hosts. Taxonomically classified within the *Stramenopiles*, this group predominantly comprises free-living, flagellated or ciliated unicellular organisms (Yason et al. 2019). Distinct from its kin, *Blastocystis* is an obligate parasite, thriving in anaerobic conditions and propagating through the faecal-oral route (Sheela et al. 2020). *Blastocystis* has the capacity to colonise the human intestinal epithelium, with severe infections that may manifest as gastrointestinal symptoms including diarrhoea, abdominal distension, anorexia, nausea, and vomiting. Furthermore, the emerging research suggests *Blastocystis* may act as a beneficial commensal. The study has demonstrated that colonisation with the ST4 subtype can positively modulate the host gut microbiota and immune responses, specifically Th2 and Treg cells (Deng et al. 2022). Despite its suggested pathogenicity, *Blastocystis* is frequently detected in asymptomatic individuals, leading to a consensus that most subtypes are likely commensal rather than pathogenic (Castaneda et al. 2020). *Blastocystis* sp. exhibits a broad global distribution, with a reported presence and high prevalence in diverse regions including northern Argentina, Japan, and Thailand (Candela et al. 2021). However, *Blastocystis* infections have also been associated with gastrointestinal and nutritional disorders in both low-income, developing, and developed countries (Chen et al. 2021).

To date, 44 subtypes of *Blastocystis* have been identified based on polymorphisms within the small subunit ribosomal RNA (*SSU rRNA*) gene. However, 38 subtypes (ST1–ST17, ST21, ST23–ST38, ST40 and ST42–ST44) are currently recognised as valid, conforming to the established criteria for unique subtype designation (Heydarian et al. 2024). Among these, ST1–ST4 are the most prevalent in humans, collectively accounting for over 90% of human infections (Deng and Tan 2022). Notably, the geographical distribution of ST4 exhibits significant variation. It is predominantly reported in Europe, but demonstrates lower prevalence in South America, Africa, and Asia.

*Sciurus vulgaris* and its subspecies, *Sciurus vulgaris exalbidus,* are widely distributed across Eurasia. In China, these squirrels are increasingly maintained as exotic companion animals, facilitated by their adaptability to captive conditions and frequent proximity to humans. However, their role as potential reservoirs for zoonotic pathogens remains understudied. Notably, *Sciurus vulgaris* populations in urban and peri-urban areas of Chengdu frequently interface with human habitats, thereby raising concerns about cross-species pathogen transmission.

*Blastocystis* infections have been documented in residents of over 12 provinces and municipalities (Chai et al. 2020). While nine subtypes (ST1-5, ST7-8, ST13, and ST17) have been identified in rodents, with ST4 being the most prevalent (Liu et al. 2021), information regarding *Blastocystis* infections in Chinese pet squirrels remains sparse (Song et al. 2021). This study aims to address this gap by evaluating the prevalence and subtypes of *Blastocystis* in *Sciurus vulgaris* and *Sciurus vulgaris exalbidus* across China’s Sichuan province, thereby enhancing our understanding of the zoonotic transmission risk of this organism.

## MATERIAL AND METHODS

### Ethical statement

This study was performed in accordance with the recommendations of the Guide for the Care and Use of Laboratory Animals of the Ministry of Health, China. Before the initiation of experiments, the protocol of the current study was reviewed and approved by the Institutional Animal Care and Use Committee of the Sichuan Agricultural University under a permit (Approval No. SYXK 2019-187). No animals were harmed during the sampling process. Permission was obtained from the pet owners or shop managers before the collection of the faecal specimens.

### Study sites

The study was conducted in Chengdu, an area of 14 335 square kilometres in China’s Sichuan province. The samples were collected in a breeding farm in Chengdu. The farm operates under a semi-open management system, with the temperature maintained at 20–25 °C. Squirrels are kept in solitary cages and fed a standardised diet of nuts, seeds, and fresh vegetables, with water provided *ad libitum*.

### Sampling

From July to August 2021, 41 faecal samples were collected from 31 *Sciurus vulgaris* (16 males, 15 females; age range: 1–3 years) and 10 *Sciurus vulgaris exalbidus* (5 males, 5 females; age range: 1–2 years) at the farm. Squirrels were selected randomly from healthy individuals without gastrointestinal symptoms during the sampling period. Fresh faecal samples (200 mg) were collected immediately after defecation using sterile gloves, placed in labelled containers, transported on ice, and stored at –80 °C within 4 hours.

### DNA extraction

The total genomic DNA was extracted directly from faecal samples (approximately 200 mg) using an E.Z.N.A. bacterial DNA Kit (Omega Bio-tek, Norcross, GA, USA), in accordance with the procedures recommended by the manufacturer. The extracted DNA was stored at –20 °C until the polymerase chain reaction (PCR) analysis.

### Subtyping of *Blastocystis* sp.

All the DNA samples were tested for *Blastocystis* spp. and the *SSU rRNA* coding region (about 510 bp) was amplified by polymerase chain reaction. The cycle parameters and primers matched the literature (Scicluna et al. 2006), see Table 1. The amplification conditions consisted of 30 cycles of 1 min each at 94 °C, 59 °C, and 72 °C, with an additional final extension at 72 °C for 2 minutes. All the PCR reactions were performed using a 2* Pro *Taq* Master Mix (Sangon Bio Inc., Beijing, P.R. China).

**Table 1:** Primers for the *Blastocystis* used in this study

Target	Primer
RD5	5'-ATCTGGTTGATCCTGCCAGT-3'
BhRDr	5'-GAGCTTTTTAACTCAACAACG-3'

All the polymerase chain reaction tests were negative controls without the addition of DNA. The polymerase chain reaction products were electrophoresed in a 1.5% agarose gel and stained with ethidium bromide.

### Sequence analysis

All the positive PCR products were directly sequenced on an ABI PRISMTM 3730 DNA Analyser (Applied Biosystems, Foster, CA, USA), using a BigDye Terminator v3.1 Cycle Sequencing kit (Applied Biosystems, Foster, CA, USA). The nucleotide sequences obtained in the present study were subjected to BLAST (http://www.ncbi.nlm.nih.gov/blast/), aligned with each other, and analysed. Reference sequences were downloaded from GenBank (http://www.ncbi.nlm.nih.gov). The sequences were aligned using Clustal X 2.0 (http://www.clustal.org/) to determine the *Blastocystis* sp. subtype. The nucleotide sequences generated in the present study were deposited in GenBank.

### Phylogenetic analyses

To assess the genetic relationships of the *Blastocystis* genotypes in this study with the sequences from GenBank identified in previous studies, a phylogenetic analysis was performed by constructing a neighbour-joining tree using MEGA v5 software (http://www.megasoftware.net/). The evolutionary distances were calculated using the Kimura 2-parameter model. Undefined positions were removed from the alignment prior to phylogenetic analysis, and the alignment was trimmed using MEGA 5 (http://www.megasoftware.net/). The reliability of the trees was assessed by a bootstrap analysis with 1 000 replicates.

## RESULTS

**Figure 1:**
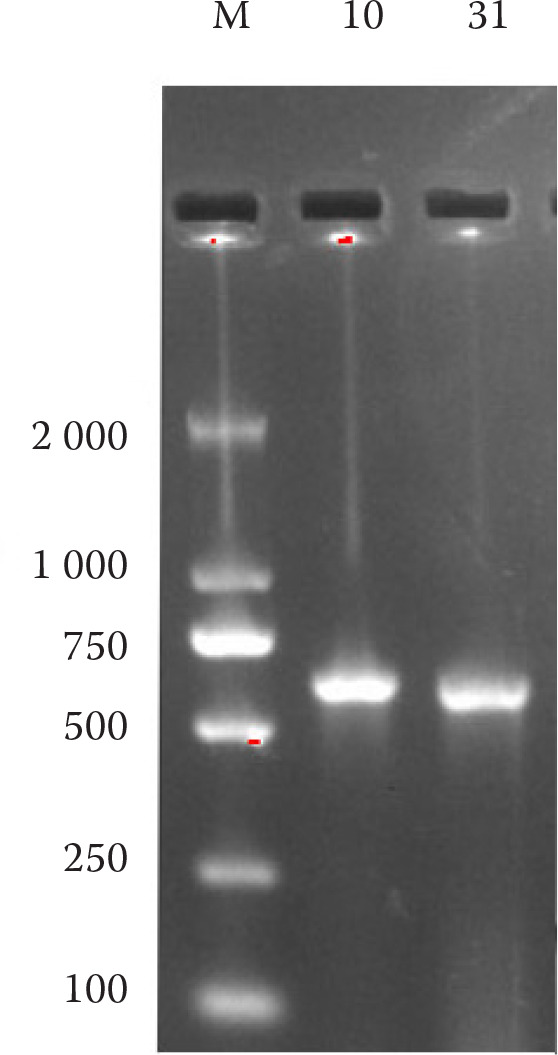
The agarose gel electrophoresis results of two out of the 41 samples in this study M: DL 2000 DNA Marker; 10: *Sciurus vulgaris exalbidus*; 31: *Sciurus vulgaris*

**Figure 2:**
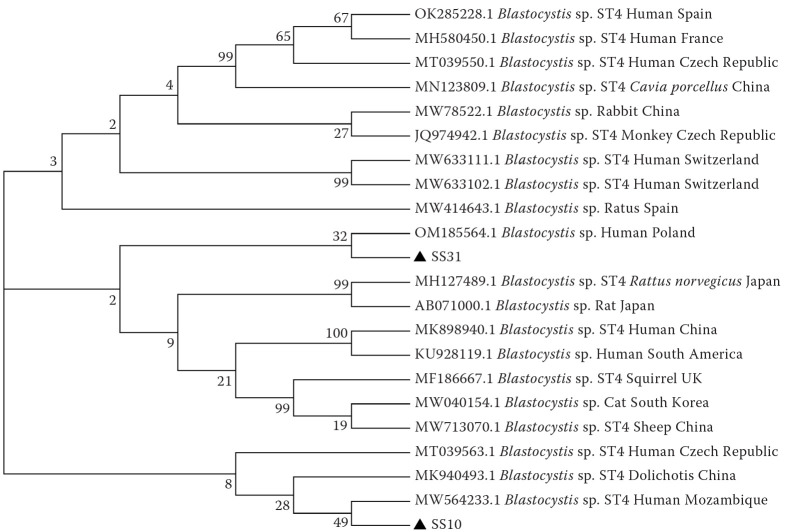
The neighbour-joining phylogenetic tree The phylogenetic relationship of the nucleotide sequences from the barcode regions of the small subunit ribosomal RNA (*SSU rRNA*) of the SS31 and SS9 strains from other *Blastocystis* sp. isolates. The neighbour-joining phylogenetic tree was constructed using MEGA 5 software. Each sequence is identified by its accession number, subtypes, origin, and country

Forty-one (41) samples were collected from Chengdu, Sichuan. Two (2) samples (4.88%) were identified as *Blastocystis*-positive by PCR (Figure 1). The subtypes were successfully sequenced, and two *Blastocystis*-positive samples were identified as ST4 based on the phylogenetic tree (Figure 2). According to the statistical analysis, the infection rate of *Sciurus vulgaris exalbidus* (10%) was higher than that of *Sciurus vulgaris* (2.7%).

The *SSU rRNA* sequences from two isolated strains (strains SS31 and SS9) were compared with 20 *Blastocystis* sp. isolates. The sequences obtained in this study showed high identity with the reference sequences of *Blastocystis* sp. in GenBank. Strain SS31 clustered together with *Blastocystis* sp. isolated from a human sample in Poland. Strain SS9 clustered together with the *Blastocystis* sp. ST4 strain that was isolated from a human sample in Mozambique (Figure 2).

## DISCUSSION

Extensive research on the molecular epidemiology of *Blastocystis* in livestock has documented transmission patterns and zoonotic significance (Deng et al. 2021). However, studies on the *Blastocystis* prevalence in squirrels remain limited. Research studies from China, Japan, Korea, Malaysia, and Mexico indicate *Blastocystis* infections in various rodent species, showing prevalence rates ranging from 4.84% to 60.37% (Katsumata et al. 2018; Martinez-Hernandez et al. 2020; Liu et al. 2022). In the present study, the PCR amplification of the *SSU rRNA* gene barcode region revealed a *Blastocystis* prevalence of 4.88% (2/41) in squirrels from Chengdu, China. This detection rate is notably lower than those reported in wild rodents from Japan (20%) (Masuda et al. 2021) and brown rats in Malaysia (45.9%) (Farah Haziqah et al. 2018). It has been reported that geographical and environmental factors might influence the prevalence of *Blastocystis* in both animals and humans. Beyond geography, variations may arise from the host age, origin, health status, and diagnostic methodologies (Liao et al. 2020). Notably, the low prevalence observed here may reflect rigorous hygiene practices, as squirrels were maintained in sanitised cages and provided with clean food and water (Chai et al. 2020).

*Blastocystis* spp. are globally prevalent intestinal parasites in humans and diverse animal hosts. Previous studies have shown that the four subtypes, ST1–ST4, have the highest prevalence rate in humans (more than 90%) (Alfellani et al. 2013). Among these, the ST4 subtype predominates in rodents such as brown rats in Japan and Polynesian rats in Indonesia (Katsumata et al. 2018). Consistent with broader regional patterns, the ST4 predominance has also been documented in China’s rodent populations (2022), which aligns with the results of this study. In addition, ST4, as a classification of *Blastocystis* protozoa, has a special geographical distribution and is most influenced by the geography and lifestyle. The existing studies have confirmed that *Blastocystis* transmission can occur between domestic animals and their handlers (Lepczynska et al. 2021). In this study, the phylogenetic analysis demonstrated that the ST4 clustered closely with human-derived sequences from Poland and Mozambique. While this suggests a potential zoonotic overlap, direct evidence of cross-species transmission requires further investigation. Importantly, ST4 has been documented in humans in multiple Chinese provinces (Deng et al. 2019; Gong et al. 2019), underscoring the need to monitor captive animals in high-contact environments. However, the small sample size (*n* = 41) and single geographic origin (Chengdu) restrict the generalisability of our findings. Future studies should expand sampling to diverse regions, include longitudinal health assessments, and incorporate metagenomic approaches to elucidate the transmission dynamics between squirrels, humans, and domestic animals.

Our study identified a 4.88% prevalence of the zoonotic ST4 subtype of *Blastocystis* in Chengdu’s squirrel population, emphasising the need for integrated surveillance and control measures to prevent cross-species transmission. The findings not only contribute to the epidemiological understanding of *Blastocystis* but also highlight the importance of considering animal reservoirs in public health strategies. Future research should expand on these results with a broader range of hosts and environments to provide information to develop effective mitigation strategies.
